# Alopecia Risk With GLP‐1 Receptor Agonists: A Disproportionality Analysis Using the FDA Adverse Event Reporting System (FAERS)

**DOI:** 10.1111/jocd.70967

**Published:** 2026-06-07

**Authors:** Aditya K. Gupta, Elizabeth Teasell, Mary A. Bamimore, Paradi Mirmirani, Mesbah Talukder

**Affiliations:** ^1^ Division of Dermatology, Temerty Faculty of Medicine University of Toronto Toronto Ontario Canada; ^2^ Mediprobe Research Inc. London Ontario Canada; ^3^ Department of Dermatology The Permanente Medical Group Vallejo California USA; ^4^ Department of Dermatology University of California San Francisco California USA; ^5^ School of Pharmacy BRAC University Dhaka Bangladesh

**Keywords:** alopecia, glucagon‐like peptide‐1 receptor agonist, obesity, pharmacovigilance

## Abstract

**Background:**

Glucagon‐like peptide‐1 receptor agonists (GLP‐1‐RAs), a class of drugs indicated for diabetes mellitus and weight control, have been implicated in alopecia. We mined data from the Food and Drug Administration (FDA) Adverse Event Reporting System (FAERS) to better understand the relationship between alopecia and GLP‐1‐RAs.

**Methods:**

Using 2016–2025 FAERS data, we conducted disproportionality analyses for 7 GLP‐1‐RAs (albiglutide, dulaglutide, exenatide, liraglutide, lixisenatide, semaglutide, and tirzepatide) and alopecia‐related adverse events (AEs); we also conducted descriptive analyses for drug‐related information.

**Results:**

Semaglutide had the highest number of reports (*n* = 697); furthermore, signals were identified with this GLP‐1‐RA in 2022 (reporting odds ratio (ROR) = 1.43, 95% confidence interval (CI): 1.15–1.77), and 2024 (ROR = 1.55, 95% CI: 1.35–1.77). We found that 25% and 18% of reports with semaglutide documented positive dechallenge and positive rechallenge, respectively.

**Conclusions:**

Our work, at the time of our pharmacovigilance study, is the first to include the latest FAERS data in disproportionality analyses for GLP‐1‐RAs and alopecia. Our descriptive analyses of salient drug‐related phenomena (including rechallenge and dechallenge information) have never been reported elsewhere. Our findings merit further monitoring of GLP‐1‐RAs for hair loss.

## 1 Introduction

Alopecia, the clinical term for hair loss, can be a cause or consequence of disturbed health [1, 2]. Despite being deemed a clinically benign cosmetic concern, various studies suggest that alopecia negatively affects mental health [2, 3]. On the other hand, this condition has recently been listed as a potential consequence of consuming glucagon‐like peptide‐1 receptor agonists (GLP‐1‐RAs) [4], a class of agents indicated for weight loss, a phenomenon that is—also like hair loss—of clinical and cosmetic significance [5, 6, 7].

In July 2023, the Food and Drug Administration (FDA) flagged alopecia as a potential untoward effect of GLP‐1‐RAs [4]. Furthermore, numerous studies have investigated the link between ‘the two losses’ [6, 7, 8, 9, 10]. The connection between hair loss and drugs for weight loss had been documented in the literature as early as 2021, the year Wadden and colleagues [11] published their randomized study (i.e., the STEP 3 Randomized Clinical Trial) where alopecia was listed as an adverse event of semaglutide use. We mined data from the FDA Adverse Event Reporting System (FAERS) to conduct disproportionality analyses for GLP‐1‐RAs and alopecia. We attempted to address knowledge gaps by: using the most up‐to‐date FAERS datasets and identifying patient‐ and drug‐related factors that could be correlated with reporting of alopecia and GLP‐1‐RAs.

## 2 Methods

Our research was carried out in alignment with the guidelines outlined in *REporting of A Disproportionality analysis for drUg Safety signal detection using individual case safety reports in PharmacoVigilance* (READUS‐PV) [12]. All statistical analyses were performed using *R* software (version 4.3.2) [13].

Through FAERS, spontaneously reported data (SRD) is freely accessible—and SRD refers to a repository of voluntarily reported adverse events (AEs). Throughout the literature, inference making for a drug's safety can be based on disproportionality analyses of SRD; ‘signal detection’ occurs when a statistically significant correlation is identified between a drug and the occurrence of an AE within this type of data source. In FAERS, AEs are described as preferred terms (pt) of the Medical Dictionary for Regulatory Activities (MedDRA) classification system.

The observation period for our study spanned about a decade (2016 to 2025), utilizing data from the first quarter of 2016 to the third quarter of 2025. The FAERS database consists of data that is organized across 7 files—each of which is published on a quarterly basis; for this study, we only needed data from 4 of the 7 files, namely, the DEMO, REAC, DRUG, and INDI datasets.

We consulted the literature to inform various aspects of our data mining, including deduplication. Duplicate reports were identified and removed well before disproportionality analyses commenced. Reports sharing the same CASEID were considered potential duplicates. For cases with multiple entries, the most recent report version was retained in accordance with FDA‐recommended procedures by using the highest PRIMARYID value. Additionally, duplicate case reports were further screened based on CASEID, event date, age, sex, and reporting country to ensure retention of the most recent and complete case version [14, 15].

The reporting odds ratio (ROR), along with its corresponding 95% confidence interval (CI), is a metric for signal detection—and is based on the foundational work of van Puijenbroek et al. (2002) [14]. When a signal is detected, the lower bound of the ROR's 95% CI is above 1 (which also translates to a *p*‐value of less than 0.05, given an alpha of 5%).

After deduplication, we also characterized patient‐ and drug‐related information concerning reports of alopecia and GLP‐1‐RAs. Information regarding age, sex, and weight was sourced from the DEMO dataset, while AEs were detailed in the REAC file. Indications for drug use were found in the INDI file, and information about a drug's dose came from the DRUG file. The drug‐related details we analyzed also included dechallenge/rechallenge information. Dechallenge and rechallenge information were extracted from the FAERS DRUG dataset using the variables DECHAL and RECHAL, respectively. Dechallenge describes the outcome following discontinuation of the suspected drug, whereas rechallenge reflects the outcome after re‐administration of the drug. DECHAL and RECHAL were categorized as positive, negative, unknown, or missing. A positive dechallenge indicated improvement or resolution of the AE after drug discontinuation, while a positive rechallenge indicated recurrence of the AE following re‐exposure to the suspected drug [15]. Missing or unknown values were not imputed; reports with missing values were excluded from proportion calculations. In other words, proportions were calculated only among reports with available dechallenge or rechallenge data. For example, if 697 semaglutide‐associated reports were identified, but only 97 contained rechallenge information and 17 of these reported a positive rechallenge, the proportion of positive rechallenge was calculated using 97 rather than 697 as the denominator.

We conducted disproportionality analyses of 7 GLP‐1‐RAs (i.e., albiglutide, dulaglutide, exenatide, liraglutide, lixisenatide, semaglutide, and tirzepatide) for the following 7 alopecia‐related AEs (as per the pts. of the MedDRA system): ‘alopecia’, ‘alopecia areata’, ‘alopecia universalis’, ‘alopecia totalis’, ‘androgenetic alopecia’, ‘non‐scarring alopecia’, and ‘diffuse alopecia’. All 7 MedDRA pts. were intentionally included in the initial search to ensure a comprehensive and systematic capture of potentially relevant alopecia‐related reports within FAERS. Although certain pts., including ‘alopecia universalis’ and ‘non‐scarring alopecia’, yielded zero reports in the final dataset, they were retained to communicate transparency of our methodology.

## 3 Results

### 3.1 Disproportionality Analyses

We identified a total of 1941 reports—across 1929 cases—documenting the occurrence of alopecia‐related AEs with use of GLP‐1‐RAs, between 2016 to 2025 in FAERS; given that very few cases (i.e., patients) reported more than one AE, the number of ‘reports’ (i.e., 1941) was slightly higher than the number of cases (i.e., 1929). We found 697, 620, 328, 282, 9 and 5 reports of alopecia‐related AEs with semaglutide, tirzepatide, liraglutide, dulaglutide, albiglutide, and lixisenatide, respectively (Tables 1 and 2). For some reports, information on the characteristics was missing; hence, where applicable, summary estimates are based on non‐missing information—as explained earlier. We did not find any reports with exenatide—nor for ‘non‐scarring alopecia or ‘alopecia universalis' (Table 1).

**TABLE 1:** Reporting odds ratio—and corresponding 95% confidence interval—from disproportionality analyses with data from United States Food and Drug Administration Adverse Event Reporting System for alopecia‐related adverse events and glucagon–like peptide–1 receptor agonist. Reports that constitute significant results (i.e., signals) are placed on top—with non‐significant results placed below.

Drug	Type of hair loss (as per the MedDRA preferred term)	Number of Cases	Lower bound of 95% CI of ROR	ROR	Upper bound of 95% CI of ROR	Year
**Significant**						
Tirzepatide	Alopecia	324	1.86	2.08	2.32	2025
Semaglutide	Alopecia	218	1.35	1.55	1.77	2024
Tirzepatide	Alopecia	204	1.36	1.56	1.79	2024
Semaglutide	Alopecia	85	1.15	1.43	1.77	2022
**Non‐significant**						
Semaglutide	Alopecia	183	0.96	1.11	1.28	2025
Dulaglutide	Alopecia	24	0.5	0.75	1.12	2025
Liraglutide	Alopecia	18	0.69	1.1	1.75	2025
Semaglutide	Diffuse alopecia	2	—	—	—	2025
Tirzepatide	Alopecia areata	2	—	—	—	2025
Tirzepatide	Androgenetic alopecia	2	—	—	—	2025
Semaglutide	Alopecia areata	1	—	—	—	2025
Semaglutide	Androgenetic alopecia	1	—	—	—	2025
Tirzepatide	Diffuse alopecia	1	—	—	—	2025
Dulaglutide	Alopecia	38	0.65	0.89	1.23	2024
Liraglutide	Alopecia	18	0.62	0.99	1.56	2024
Semaglutide	Alopecia areata	3	0.63	1.98	6.15	2024
Semaglutide	Diffuse alopecia	2	—	—	—	2024
Tirzepatide	Alopecia areata	2	—	—	—	2024
Lixisenatide	Alopecia	1	—	—	—	2024
Semaglutide	Androgenetic alopecia	1	—	—	—	2024
Tirzepatide	Diffuse alopecia	1	—	—	—	2024
Semaglutide	Alopecia	101	0.88	1.07	1.3	2023
Tirzepatide	Alopecia	68	0.9	1.14	1.45	2023
Dulaglutide	Alopecia	29	0.48	0.69	1	2023
Liraglutide	Alopecia	18	0.54	0.86	1.37	2023
Semaglutide	Alopecia areata	2	—	—	—	2023
Dulaglutide	Alopecia areata	1	—	—	—	2023
Liraglutide	Alopecia totalis	1	—	—	—	2023
Semaglutide	Diffuse alopecia	1	—	—	—	2023
Tirzepatide	Alopecia areata	1	—	—	—	2023
Dulaglutide	Alopecia	33	0.55	0.77	1.08	2022
Liraglutide	Alopecia	17	0.53	0.85	1.37	2022
Tirzepatide	Alopecia	9	0.3	0.58	1.11	2022
Liraglutide	Alopecia totalis	1	—	—	—	2022
Lixisenatide	Alopecia	1	—	—	—	2022
Semaglutide	Alopecia areata	1	—	—	—	2022
Semaglutide	Alopecia totalis	1	—	—	—	2022
Tirzepatide	Diffuse alopecia	1	—	—	—	2022
Semaglutide	Alopecia	47	0.74	0.98	1.3	2021
Dulaglutide	Alopecia	34	0.42	0.59	0.83	2021
Liraglutide	Alopecia	26	0.66	0.97	1.43	2021
Albiglutide	Alopecia	1	—	—	—	2021
Dulaglutide	Alopecia areata	1	—	—	—	2021
Liraglutide	Alopecia	43	0.8	1.08	1.46	2020
Semaglutide	Alopecia	36	0.66	0.91	1.26	2020
Dulaglutide	Alopecia	32	0.39	0.55	0.78	2020
Albiglutide	Alopecia	1	—	—	—	2020
Liraglutide	Alopecia	56	0.76	0.99	1.29	2019
Dulaglutide	Alopecia	29	0.27	0.39	0.56	2019
Semaglutide	Alopecia	4	0.06	0.16	0.42	2019
Lixisenatide	Alopecia	2	—	—	—	2019
Liraglutide	Alopecia	67	0.69	0.88	1.12	2018
Dulaglutide	Alopecia	31	0.31	0.44	0.62	2018
Semaglutide	Alopecia	2	—	—	—	2018
Albiglutide	Alopecia	1	—	—	—	2018
Dulaglutide	Alopecia areata	1	—	—	—	2018
Lixisenatide	Alopecia	1	—	—	—	2018
Liraglutide	Alopecia	30	0.68	0.97	1.39	2017
Dulaglutide	Alopecia	15	0.26	0.43	0.71	2017
Albiglutide	Alopecia	3	0.16	0.5	1.56	2017
Dulaglutide	Diffuse alopecia	1	—	—	—	2017
Liraglutide	Alopecia	33	0.82	1.16	1.63	2016
Dulaglutide	Alopecia	12	0.38	0.66	1.17	2016
Albiglutide	Alopecia	3	0.04	0.12	0.39	2016

*Note:* We mined the United States Food and Drug Administration Adverse Event Reporting System to analyze 7 glucagon–like peptide–1 receptor agonists (i.e., Albiglutide, Dulaglutide, Exenatide, Liraglutide, Lixisenatide, Semaglutide, and Tirzepatide) and the following 7 alopecia‐related adverse events as per MedDRA classification: ‘alopecia’, ‘alopecia areata’, ‘alopecia totalis’, ‘androgenetic alopecia’, ‘diffuse alopecia’, ‘non‐scarring alopecia’, and ‘alopecia universalis’. This table only presents from reports where any of the 7 AEs was reported with one of the alopecia‐related AEs. Exenatide is not presented in the table because we found no reports with Exenatide across our observation period (i.e., 2016–2025). The rows with bold text and shaded cells each corresponds to reports for which a signal was detected as per the van Puijenbroek et al.'s (2002) formulae.

Abbreviations: MedDRA, Medical Dictionary for Regulatory Activities; ROR, Reporting odds ratio.

**TABLE 2:** A summary of patient‐related and drug‐related characteristics from reports of alopecia‐relted AEs and GLP–1–RAs within the FAERS database from 2016 to 2025 (inclusive).

Characteristic	Albiglutide, *N* = 9^a^	Dulaglutide, *N* = 282^a^	Liraglutide, *N* = 328^a^	Lixisenatide, *N* = 5^a^	Semaglutide, *N* = 697^a^	Tirzepatide, *N* = 620^a^
Gender						
Female	7 (100%)	239 (90%)	295 (94%)	4 (80%)	592 (91%)	496 (92%)
Male	0 (0%)	26 (9.8%)	18 (5.8%)	1 (20%)	58 (8.9%)	44 (8.1%)
Missing	2	17	15	0	47	80
Age, years	63.00 (4.69)	61.56 (11.76)	57.60 (11.56)	61.60 (7.64)	58.26 (13.88)	56.76 (14.42)
Missing	5	75	88	0	186	158
Weight, kilograms	75.50 (21.92)	71.52 (20.11)	75.78 (27.48)	72.47 (20.13)	70.06 (23.28)	62.35 (18.28)
Missing	5	71	78	0	175	153
Reporter's occupation						
Consumer	7 (78%)	208 (75%)	194 (60%)	4 (80%)	489 (71%)	554 (90%)
Lawyer	1 (11%)	11 (4.0%)	40 (12%)	0 (0%)	10 (1.5%)	4 (0.7%)
Other Health Professional	0 (0%)	27 (9.7%)	37 (11%)	1 (20%)	76 (11%)	32 (5.2%)
Pharmacist	1 (11%)	9 (3.2%)	11 (3.4%)	0 (0%)	23 (3.4%)	7 (1.1%)
Physician	0 (0%)	23 (8.3%)	44 (13%)	0 (0%)	86 (13%)	17 (2.8%)
Missing	0	4	2	0	13	6
Adverse event (as per MedDRA preferred term classification)						
Alopecia	9 (100%)	278 (99%)	326 (99%)	5 (100%)	682 (98%)	610 (98%)
Alopecia areata	0 (0%)	3 (1.1%)	0 (0%)	0 (0%)	7 (1.0%)	5 (0.8%)
Alopecia totalis	0 (0%)	0 (0%)	2 (0.6%)	0 (0%)	1 (0.1%)	0 (0%)
Androgenetic alopecia	0 (0%)	0 (0%)	0 (0%)	0 (0%)	2 (0.3%)	2 (0.3%)
Diffuse alopecia	0 (0%)	1 (0.4%)	0 (0%)	0 (0%)	5 (0.7%)	3 (0.5%)
Dechallenge						
Does not apply	1 (17%)	6 (4.1%)	8 (5.3%)	1 (50%)	17 (4.1%)	2 (0.4%)
Negative dechallenge	0 (0%)	8 (5.4%)	20 (13%)	0 (0%)	78 (19%)	49 (10%)
Positive dechallenge	1 (17%)	24 (16%)	37 (25%)	0 (0%)	106 (25%)	42 (8.7%)
Unknown	4 (67%)	110 (74%)	86 (57%)	1 (50%)	217 (52%)	390 (81%)
Missing	3	134	177	3	279	137
Rechallenge						
Does not apply	1 (50%)	3 (5.6%)	5 (8.5%)	0 (NA%)	41 (42%)	17 (57%)
Negative rechallenge	0 (0%)	2 (3.7%)	1 (1.7%)	0 (NA%)	2 (2.1%)	1 (3.3%)
Positive rechallenge	0 (0%)	4 (7.4%)	6 (10%)	0 (NA%)	17 (18%)	10 (33%)
Unknown	1 (50%)	45 (83%)	47 (80%)	0 (NA%)	37 (38%)	2 (6.7%)
Missing	7	228	269	5	600	590

Abbreviations: AE, adverse event; FAERS, food and drug administration adverse event reporting system, GLP–1–RA, GLUCAGON–like peptide–1 receptor agonist; MedDRA, medical dictionary for regulatory activities, *N*, number of reports.

^a^ *n* (%); mean (±standard deviation).

**FIGURE 1:**
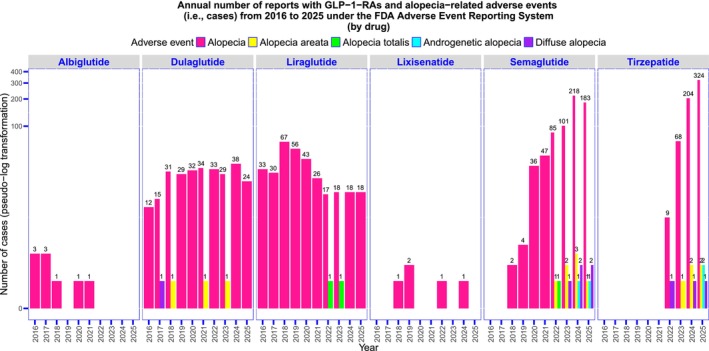
Annual number of FAERS reports involving glucagon‐like peptide‐1 receptor agonists (GLP‐1‐RAs) and alopecia‐related adverse events from 2016 to 2025, grouped by individual GLP‐1‐RA agent. Each panel corresponds to a specific GLP‐1‐RA (albiglutide, dulaglutide, liraglutide, lixisenatide, semaglutide, and tirzepatide). The x‐axis represents reporting year (2016–2025), while the y‐axis represents the number of adverse event reports (cases) displayed using a pseudo‐logarithmic transformation to improve visualization of both low‐ and high‐frequency report counts within the same figure. Colored bars represent different alopecia‐related Medical Dictionary for Regulatory Activities (MedDRA) preferred terms, including alopecia, alopecia areata, alopecia totalis, androgenetic alopecia, and diffuse alopecia. Numeric labels above bars indicate the exact number of reports for each drug–event–year combination.

**FIGURE 2:**
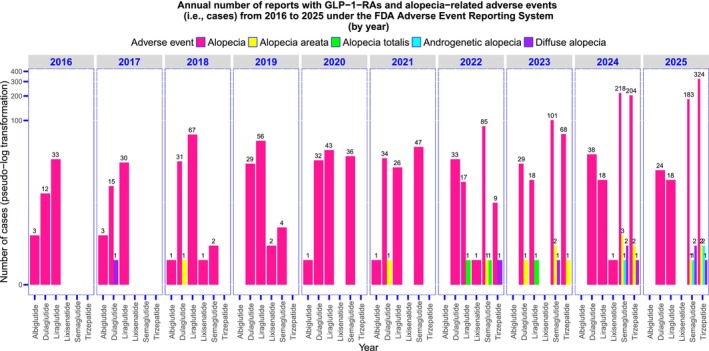
Annual distribution of FAERS reports involving glucagon‐like peptide‐1 receptor agonists (GLP‐1‐RAs) and alopecia‐related adverse events from 2016 to 2025, grouped by reporting year. Each panel corresponds to a specific calendar year, and within each panel, the number of reports is stratified by GLP‐1‐RA agent (albiglutide, dulaglutide, liraglutide, lixisenatide, semaglutide, and tirzepatide). The x‐axis displays the GLP‐1‐RA agents, whereas the y‐axis represents the number of adverse event reports (cases) presented using a pseudo‐logarithmic transformation to facilitate simultaneous visualization of sparse and highly frequent reports. Bar colors correspond to distinct alopecia‐related Medical Dictionary for Regulatory Activities (MedDRA) preferred terms, including alopecia, alopecia areata, alopecia totalis, androgenetic alopecia, and diffuse alopecia. Numeric annotations above bars denote the corresponding number of FAERS reports.

The annual number of reports with GLP‐1‐RA and alopecia‐related AEs have been presented by drug in Figure 1, and by year in Figure 2. Reporting odds ratio (and corresponding 95% CI) for each AE, in each year, is presented in Table 1—and only AEs for which there is at least 1 report are presented therein. Across all reports, we detected signals only for semaglutide and tirzepatide. Signals were detected for semaglutide in 2022 (ROR = 1.43, 95% CI: 1.15–1.77), and 2024 (ROR = 1.55, 95% CI:1.35–1.77) (Table 1). Signals were also detected for tirzepatide in 2024 (ROR = 1.56, 95% CI:1.36–1.79) and 2025 (ROR = 2.08, 95% CI:1.86–2.32) (Table 1).

### 3.2 Patient Characteristics

Patient‐related and drug‐related information across the 1941 reports have been summarized in Table 2; therein, information has been provided for patients' sex, age (in years), and weight (in kilograms); also provided is information on the reporter's occupation, dechallenge and rechallenge.

For each drug, most (i.e., over 75%) of patients were women. The average age (±standard deviation (SD)) of patients who used semaglutide and tirzepatide was 58.26 (±13.88) years and 56.76 (±14.42) years, respectively. The average weight (SD) of patients who used semaglutide and tirzepatide was 70.06 (±23.28) kilograms (kg) and 62.35 (±18.28) kg, respectively. Consumers were the most common reporter group for most drugs; consumer reporting exceeded 70% for all drugs except liraglutide (60%).

### 3.3 Drug Characteristics—Dose

General information on each drug's dose strength—as documented in the respective drugs' package insert (or prescribing information)—has been collated in Table 3. Based on the information in Table 3, our descriptive analyses for dose only included reports where dose was reported in milligrams and for ‘plausible’ strengths, because incorrect reporting of dose information is not uncommon with FAERS [16]. Therefore, some reports were excluded; for example, about a few of the 1941 reports documented a dose strength of 500 mg—and such were not included, because a 500 mg strength is non‐existent in the market. For semaglutide, the most frequent dose strength reported was 1.00 mg (number of reports = 62); the lowest and highest dose strengths were 0.25 mg (number of reports = 49), and 14 mg (number of reports = 5), respectively. For Tirzepatide, the most frequent dose strength reported was 2.50 mg (number of reports = 192); the highest and lowest dose strengths were 2.5 mg (number of reports = 192), and 15 mg (number of reports = 20), respectively.

**TABLE 3:** Information on dose strengths of commonly used GLP–1–RAs.

Drug	Trade name(s)	Dose(s)
Lixisenatide	Adlyxin	10 mcg, 20 mcg
	Lyxumia	10 mcg, 20 mcg
	Soliqua/Suliqua	5.3 mcg – 20 mcg
Exenatide	Bydureon	2 mg
	Byetta	5 mcg, 10 mcg
Tirzepatide	Mounjaro	2.5 mg, 5 mg, 7.5 mg, 10 mg, 12.5 mg, 15 mg
	Zepbound	2.5 mg, 5 mg, 7.5 mg, 10 mg, 12.5 mg, 15 mg
Semaglutide	Ozempic	0.25 mg, 0.5 mg, 1 mg, 2 mg
	Rybelsus	1.5 mg, 3 mg, 4 mg, 7 mg, 9 mg, 14 mg
	Wegovy	0.25 mg, 0.5 mg, 1 mg, 1.7 mg, 2 mg
Liraglutide	Sexenda	0.6 mg, 1.2 mg, 1.8 mg, 2.4 mg, 3 mg
	Victoza	0.6 mg, 1.2 mg, 1.8 mg
Dulaglutide	Trulicity	0.75 mg, 1.5 mg, 3 mg, 4.5 mg
Albiglutide	Tanzeum	30 mg, 50 mg

*Note:* The information collated in this table was obtained through searches through Drugs.com (https://www.drugs.com/).

Abbreviations: mcg, micrograms; mg, milligrams.

### 3.4 Drug Characteristics—Dechallenge and Rechallenge

Challenge information was available for all drugs that reported an alopecia‐related AE. As mentioned earlier, negative rechallenge refers to the absence of an AE even after a drug is re‐introduced, while positive rechallenge corresponds to the reoccurrence of an AE after re‐introduction of the drug. Negative dechallenge refers to the persistence of an AE even after the drug is withdrawn, while positive dechallenge corresponds to the disappearance of an AE when it is withdrawn [17]. We found that 19% and 10% of semaglutide and tirzepatide reports documented negative dechallenge, respectively; we found that 18% and 33% of semaglutide and tirzepatide reports documented positive rechallenge, respectively (Table 2).

### 3.5 Drug Characteristics —Indication for Use

**FIGURE 3:**
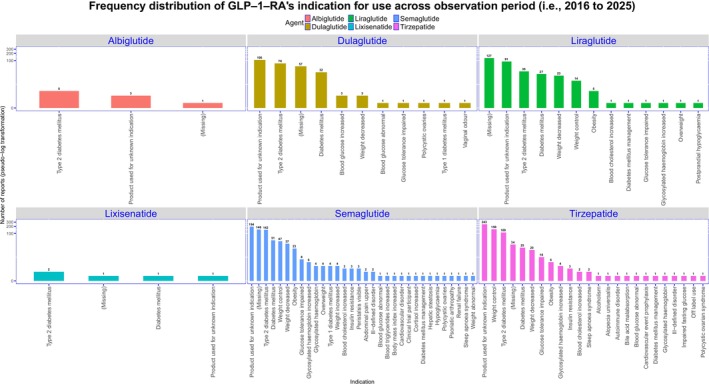
Frequency distribution of indications for use among FAERS reports involving glucagon‐like peptide‐1 receptor agonists (GLP‐1‐RAs) and alopecia‐related adverse events across the observation period (2016–2025). Each panel represents a specific GLP‐1‐RA agent (albiglutide, dulaglutide, liraglutide, lixisenatide, semaglutide, and tirzepatide). The x‐axis displays the reported indication terms extracted from FAERS, while the y‐axis represents the number of reports presented using a pseudo‐logarithmic transformation to improve visualization across a broad range of report frequencies. Colored bars correspond to individual GLP‐1‐RA agents, and numeric labels above bars indicate the exact number of reports associated with each indication term. Commonly reported indications included type 2 diabetes mellitus, obesity, weight control, diabetes mellitus, and weight decreased.

For each drug, we produced a frequency distribution pertaining to the indication for use (Figure 3). Across all drugs, diabetes mellitus and weight control were among the top 10 indications for use. We also found ‘product used for unknown indication’ among the top 10 indications for use. Even though ‘off‐label use’ and ‘product used for unknown indication’ are distinct under FAERS, the latter could be suggestive of the former, which suggests off‐label use of these drugs (Figure 3).

## 4 Discussion

To summarize our work, we used 2016 to 2025 FAERS data to investigate the relationship between GLP‐1‐RAs and alopecia by examining 7 drugs (i.e., albiglutide, dulaglutide, exenatide, liraglutide, lixisenatide, semaglutide, and tirzepatide) and 7 AEs as per the MedDRA pts. classification system (i.e., ‘alopecia’, ‘alopecia areata’, ‘alopecia totalis’, ‘androgenetic alopecia’, ‘diffuse alopecia’, ‘non‐scarring alopecia’, ‘alopecia universalis’).

Our results are consistent with market trends over time. For instance, the complete lack of alopecia‐related AE reports we identified for exenatide could reflect the decreased use of it over time (according to Diabetes UK and United Healthcare) [18, 19]; by late 2024, exenatide was completely discontinued solely for economic reasons. On the other hand, semaglutide and tirzepatide are the most sold GLP‐1‐RAs; at a time, the supply of this class of drugs could not keep up with the demand thereof [20]. In fact, Mattingly et al. (2025) had narrated how semaglutide was the first drug listed on the FDA drug shortages list in March 2022 [20]. Our analyses very well reflects this demand because, across the 6 different GLP‐1‐RAs, semaglutide had the highest reports of alopecia‐related AEs from 2021 to 2024—and second‐highest in 2025. The widespread use of the two (i.e., semaglutide and tirzepatide) have been cited numerous times in the peer‐reviewed literature [7, 21, 22, 23, 24, 25].

Our results are consistent with findings from previous studies. The results of the current study resonate with those of Singal et al., whose propensity‐score matched study examined the occurrence of hair loss with GLP‐1‐RA users with diabetes mellitus using data from TriNetX Research Network [24]. The authors' descriptive analyses, like ours, showed GLP‐1‐RA users to be majorly female—and aged, on average, in their 50's. The authors found that, compared to use of metformin (i.e., the control), the risk of developing hair loss was greater with semaglutide (hazard ratio [HR] = 4.4, 95% CI: 3.8–5.2, *p* < 0.05) and tirzepatide (HR = 5.7, 95% CI: 4.4–7.2, *p* < 0.05). Similarly, Godfrey et al. (2025) showed an association between Semaglutide and Tirzepatide using 2022 to 2023 FAERS data [9]. Though we used a much longer observation period (i.e., 10 years), results from the Godfrey et al.'s (2025) 1‐year disproportionality analyses are congruent with ours. As stated earlier, our disproportionality analyses for semaglutide produced RORs of 1.55 (*p* < 0.05) in 2024, and 1.43 (*p* < 0.05) in 2022; our disproportionality analyses for tirzepatide produced RORs of 1.56 (*p* < 0.05) in 2024, and 2.08 (*p* < 0.05) in 2025.

Though GLP‐1‐RAs have been implicated in hair loss [26], the decades of rigorous science upon which they were developed forever merit them as a well‐established therapeutic class of drugs rooted in long‐standing science [27]. Glucagon‐like peptide‐1 receptor agonists could be thought of as safe because—as Shah and Bhattacharya explicated [25]—it is about 98% identical to the body's naturally occurring GLP‐1. Notwithstanding the high resemblance, various kinds of AEs have still been linked to GLP‐1‐RAs. The resemblance and AE connection could be reconciled by ‘temporal differences’: the half‐life of native GLP‐1 in the bloodstream is very short (i.e., up to 2 min) while that of the synthesized analog is far longer (approximately 165 h) [25]. The biochemical property of a longer half‐life would be both ‘boon and bane’: by having a structure that prevents it from being quickly degraded (unlike native GLP‐1 that is subject to rapid enzymatic degradation), GLP‐1‐RAs can sustain GLP‐1's effects for longer—which consequently translates to weight control; however, the longer half‐life could simultaneously play a role in perpetuating metabolic changes that could result in untoward effects like alopecia, among other things [22].

This study has several limitations inherent to the FAERS database. As a spontaneous reporting database, FAERS is subject to underreporting, reporting bias, stimulated reporting, and incomplete or inaccurate clinical information, meaning that the number of reported adverse events may not reflect the true incidence or prevalence of alopecia‐related events among users of GLP‐1‐RAs. Although duplicate reports were systematically identified and removed prior to analysis, residual duplication cannot be entirely excluded. Furthermore, FAERS lacks reliable denominator data, thereby preventing the calculation of incidence rates or absolute risk estimates; consequently, reporting odds ratios (RORs) should be interpreted as measures of disproportionality rather than causality. Important clinical and patient‐level variables are also incompletely captured within FAERS, limiting the ability to adjust for potential confounders such as underlying autoimmune or dermatologic conditions, nutritional deficiencies, hormonal disorders, concomitant medications, and disease severity, all of which may independently contribute to risk of developing hair loss. In addition, temporal associations reported in FAERS do not establish a causal relationship between GLP‐1‐RA exposure and alopecia‐related AEs. Nevertheless, despite these limitations, FAERS remains a valuable pharmacovigilance resource for the early detection of potential safety signals and hypothesis generation, particularly for rare or emerging AEs that may not be readily identified in premarketing clinical trials [28].

Though causation cannot be established with observational data, the information the current study presented on dechallenge and rechallenge, from a decade's worth of FAERS data, could indirectly contribute to causal inferences—and our study is the first to present such information. Our study would also be the first to strongly support the possibility of a ‘media effect’: we cannot rule out that the reporting of semaglutide and tirzepatide could be attributable to media attention around the two. We observed a jump in reporting around the period the FDA flagged alopecia as an AE of GLP‐1‐RAs (Figure 1); as mentioned earlier, the FDA sent out the warning in 2023 and we found far more reports after 2022 than before 2022 for semaglutide and tirzepatide.

## Author Contributions

Conception of the manuscript was done by A.K.G. Data analysis was performed by E.T. and M.A.B. The manuscript was drafted by A.K.G., M.T., E.T., and M.A.B. The manuscript was substantively edited and revised by A.K.G., M.T., M.A.B., and P.M.

## Funding

The authors have nothing to report.

## Ethics Statement

The authors have nothing to report.

## Consent

The authors have nothing to report.

## Conflicts of Interest

The authors declare no conflicts of interest.

## Data Availability

The data that support the findings of this study are available from the corresponding author upon reasonable request.
